# Accelerated Corneal Endothelial Cell Loss after Phacoemulsification in Patients with Mildly Low Endothelial Cell Density

**DOI:** 10.3390/jcm10112270

**Published:** 2021-05-24

**Authors:** Hung-Chi Chen, Chen-Wei Huang, Lung-Kun Yeh, Fang-Chi Hsiao, Yi-Jen Hsueh, Yaa-Jyuhn James Meir, Kuan-Jen Chen, Chao-Min Cheng, Wei-Chi Wu

**Affiliations:** 1 Department of Ophthalmology, Chang Gung Memorial Hospital, Taoyuan 333423, Taiwan; lkyeh@ms9.hinet.net (L.-K.Y.); t6612@seed.net.tw (Y.-J.H.); cgr999@gmail.com (K.-J.C.); weichi666@gmail.com (W.-C.W.); 2 Department of Medicine, College of Medicine, Chang Gung University, Taoyuan 333323, Taiwan; wayne70521218@gmail.com; 3 Center for Tissue Engineering, Chang Gung Memorial Hospital, Taoyuan 333423, Taiwan; 4 Department of Biomedical Sciences, College of Medicine, Chang Gung University, Taoyuan 333323, Taiwan; hercats1@gmail.com; 5 Institute of Biomedical Engineering, National Tsing Hua University, Hsinchu 300044, Taiwan; chaomin@mx.nthu.edu.tw

**Keywords:** cataract, phacoemulsification, corneal endothelial cells

## Abstract

By evaluating preoperative endothelial cell density (ECD), ECD loss after phacoemulsification can be predicted. In this retrospective cross-sectional study, we compared outcomes of phacoemulsification with different levels of preoperative ECD. Three-hundred-and-fifty-three patients aged between 18 and 90 years received phacoemulsification at Chang Gung Memorial Hospital. Age (*p* = 0.003), preoperative logMAR (*p* = 0.048), cataract grade (*p* = 0.005), preoperative ECD (*p* < 0.001), operation time (*p =* 0.043), phacoemulsification time (*p =* 0.001), and phacoemulsification energy (*p <* 0.001) were significantly associated with postoperative ECD change (%). Patients were divided into three groups according to preoperative ECD levels. Level of ECD, coefficient of variation (CV), cell hexagonality (HEX), central corneal thickness (CCT), visual acuity, underlying diseases, and complications were analyzed. With regard to groups, 29, 71, and 252 patients were respectively allocated into the markedly low (group A; ECD below 1000 cells/mm^2^), mildly low (group B; ECD between 1000 to 2000 cells/mm^2^), and normal (group C; ECD above 2000 cells/mm^2^) ECD level groups. The highest CV (40.8 ± 13.9%; *p <* 0.001) and lowest HEX (58.4 ± 14.6%; *p <* 0.001) were found in group A. Significant ECD loss was found in group B (28.9 ± 9.2%) as compared to group A (19.9 ± 5.4%) and C (15.0 ± 12.0%) (*p <* 0.001). No significant differences were found with regard to changes in CV (*p =* 0.941), HEX (*p =* 0.937), CCT (*p =* 0.346), and logMAR (*p =* 0.557) among the three groups. In conclusion, preoperative ECD level could be a novel predictive value for postoperative cell loss, which was the most prominent in mildly low ECD level group. Less phacoemulsification energy, earlier surgical intervention, or novel topical medications could be suggested for patients with an ECD range from 1000 to 2000 cells/mm^2^.

## 1. Introduction

Human corneal endothelial cells (HCECs) are responsible for maintaining corneal transparency through regulating the hydration of the corneal stroma. Reduced HCEC, mostly due to previous intraocular surgery, trauma, corneal endotheliitis, and corneal endothelial dystrophy may cause decompensation, leading to corneal edema with visual impairment [1,2,3,4]. Phacoemulsification for cataracts is one of the most commonly performed surgical procedures, and decreased corneal endothelial cell density (ECD) is a major postoperative complication, resulting in compromised vision [5,6]. The known mechanisms include cornea distortion, intraocular lens contact, nuclear fragment contact, free radical production, etc. [3,4,7]. Even with advanced modern techniques, a decline of ECD to approximately 4.2–16.7% is still inevitable [3,4,8]. Thus, identifying the risk factors for increased loss of HCEC is mandatory for optimizing the outcomes of phacoemulsification.

Previously, several preoperative risk factors have been identified to increase ECD loss after phacoemulsification, such as hardness of nucleus, shorter axial length, or presence of diabetes mellitus [4,9,10]. Besides, higher consumed ultrasonographic energy, longer aspiration time spent, larger volume of balanced salt solution infused, and posterior capsular rupture during surgery all cause a significant decline in ECD [3,4,8,11]. Although no significant correlation has been detected between preoperative ECD level and postoperative HCEC loss in populations with markedly low ECD levels (ECD < 1000 cells/mm^2^) [3,4] and normal ECD levels [11,12] (ECD > 2000 cells/mm^2^), little is known about the scenarios in patients with mildly low ECD levels (1000–2000 cells/mm^2^).

In this study, by comparing three groups of patients with different levels of preoperative corneal ECD, i.e., the markedly low ECD level (<1000 cells/mm^2^), mildly low ECD level (1000–2000 cells/mm^2^), and normal ECD level (>2000 cells/mm^2^) groups, we demonstrated significantly more HCEC loss in patients with mildly low ECD than those in the other two groups.

## 2. Patients and Methods

### Ethnic Declaration and Participants

The study was approved and monitored by the Institutional Review Board at Chang Gung Memorial Hospital and adhered to the declaration of Helsinki. A retrospective non-randomized cross-sectional study was conducted by chart review in a 3-year interval. The inclusion criteria of the study group included patients with: (1) history of phacoemulsification with intraocular lens (IOL) implantation due to a cataract; (2) a postoperative follow-up period of more than 3 months; (3) available corneal endothelial examination information both preoperatively and postoperatively; (4) age > 40 years old. On the other hand, the exclusion criteria included patients with: (1) missing data with regard to specular microscope examination; (2) history of ocular trauma; and (3) previous intraocular surgery.

## 3. Surgical Procedure

The surgical procedures were performed by an experienced surgeon (Chen, HC) using standard soft-shell techniques [13,14,15]. In brief, a side-port and a 2.65 mm main wound at the temporal side were made at the limbus. Continuous curvilinear capsulorhexis was performed with grasping forceps after viscoelastic substances (Duovisc, Alcon, Fort Worth, TX, USA) were injected into the anterior chamber for space maintenance. The lens nucleus was removed through phacoemulsification using the soft-shell technique, while residual cortex was removed through automated irrigation and aspiration. The phaco device settings for the Infiniti device (Alcon, Fort Worth, TX, USA) were as follows: bottle height, 80 to 100 cm; vacuum pressure, 250 mm Hg; flow rate, 25 mL/min; and longitudinal ultrasound power, 50%. A foldable hydrophobic acrylic IOL (AAB00, AMO, Santa Ana, CA, USA) was implanted in the bag with a cartridge and an injector. After complete removal of viscoelastic materials, the clear corneal incisions were hydro-sealed. After surgery, the use of an overnight pressure patch was followed by daily topical betamethasone–tobramycin solution 4 times a day for 1 month.

## 4. Ophthalmic Examinations

All the ophthalmic examinations were performed in both eyes within 1 month preoperatively. Measurements of corneal endothelial morphology and central corneal thickness, accompanied by other routine ophthalmic exams such as visual acuity and intraocular pressure were documented for as long as 6 months after surgery. Visual acuity (VA) and best corrected visual acuity (BCVA) were measured by the Snellen chart, with measurements converted to logMAR for analysis. Intraocular pressure (IOP) and biometry including spherical equivalent, corneal curvature, CCT, and axial length were collected via a pneumatonometer (Canon, TX-10, Canon Corporation, Tokyo, Japan), autorefractometer (KR-7000, Tokyo, Japan), and IOLMaster (Carl Zeiss Meditec, Inc., Dublin, CA, USA). We adopted the energy consumed in phacoemulsification to replace cataract grade in this study, which is a numerical but not nominal measure, as recently reported [16]. In addition, a non-contact in vivo specular microscope (CEM-530, Nidek, Gamagori, Japan) with nominal magnification of 400× was used to take photos of the corneal endothelium for evaluation. The technician measured toward the central cornea once, and extra attempts were made if the image quality could not reach the required threshold. After measurements, data were transmitted to the built-in software program, which immediately calculated the mean cell area, standard deviation, coefficient of variation, ECD, and percentage of hexagonal cells. All the eyes in the study group, the control group, and the contralateral group received identical examinations. If VA and BCVA values were below 0.01 and were recorded as a semiquantitative scale, the value of LogMAR was set at 1.85 for counting fingers, 2.3 for hand motion, 2.6 for light perception, and 2.9 for no light perception according to the experience of Holladay and the University of Freiburg study group results [17,18].

## 5. Statistical Analysis

All of the statistical data were analyzed using SPSS 20 (SPSS Inc. Chicago, IL, USA). The Kolmogorov–Smirnov test was used to check multivariate normality. A *p*-value < 0.05 was used to define normality. Pearson’s chi-squared test was applied to measure the association between ECD changes and age, gender, operation eye, preoperative IOP, preoperative VA, cataract, ocular and systemic cormobilities, preoperative HCEC status, and OP conditions. The impact of variables’ on ECD change was evaluated by multiple linear regression analysis. One-way analysis of variance with the Scheffe test was used to evaluate the postoperation parameters, HCEC variance, HCEC hexagonality, ECD loss, and postoperative CCT change among the markedly low ECD group, mild low ECD group, and normal ECD group. A *p*-value < 0.05 was regarded as a significant difference using a two-tailed probability at 95% confidence intervals.

## 6. Results

Patients between the ages of 41 and 90 years who received phacoemulsification at Chang Gung Memorial Hospital were included in this retrospective study. During the study period of 2 years, 352 patients with cataract received phacoemulsification with the aforementioned method. To further analyze the postoperative outcomes affected by different preoperative ECD levels, the patients were divided into the markedly low ECD level group (group A; ECD below 1000 cells/mm^2^), mildly low ECD level group (group B; ECD between 1000 to 2000 cells/mm^2^), and normal ECD level group (group C; ECD above 2000 cells/mm^2^), respectively. The Kolmogorov–Smirnov (K-S) test showed that the normal distribution of the preoperative ECD of 352 patients was not present (*p <* 0.001). Nevertheless, the K-S test showed that a normal distribution (*p =* 0.200, *p =* 0.200) was present in groups A and C, but not in group B (*p* = 0.014), suggesting that group B had different characteristics from the other two groups. (Figure S1 and Table S1).

## 7. Demographic and Clinical Characteristics of the Preoperative Parameters

There were 352 cases in this study. The mean age was 69 years, ranging from 41 years to 90 years. Overall, 143 males and 209 females were included. The mean preoperative logMAR recorded in 352 patients was 1.05 ± 0.47. The cataract grade was mostly grade II (90.9%). The systemic comorbidities of hypertension and diabetes were present in 133 (37.8%) and 107 (30.4%) patients. The mean preoperative ECD was 2268.7 ± 679.5 cells/mm^2^ in the 352 patients. The coefficient of variation and cell hexagonality values were 34.2 ± 11.1% and 63.8 ± 10.0%, respectively. The preoperative mean CCT was 553.3 ± 34.0 μm (Table 1).

There were significant differences with regard to preoperative ECD (*p <* 0.001), CV (*p <* 0.001), HEX (*p <* 0.001), postoperative ECD (*p <* 0.001), CV (*p <* 0.001), HEX (*p =* 0.001), and percentage and number of postoperative ECD changes (*p <* 0.001), as well as Fuchs’ endothelial dystrophy (*p <* 0.001). There were no significant differences in age, gender, selected eye, preoperative visual acuity, preoperative intraocular pressure, cataract grade, phacoemulsification energy, anterior chamber depth, ocular and systematic comorbidities including glaucoma, hypertension and diabetes, and preoperative CCT among the groups A, B, and C in one-way ANOVA.

The mean ECD in groups A, B, and C appeared significantly different (*p <* 0.001). As for the cell morphology, group C showed a significantly lower coefficient of variation than groups A and B (*p <* 0.001) and significantly higher cell hexagonality than groups A and B (*p <* 0.001). In total, 32 (9.1%) patients had corneal dystrophy, including 29 patients with Fuchs’ endothelial dystrophy, 1 with granular dystrophy, and 1 with unspecific corneal dystrophy in group B, and 1 with basement membrane dystrophy in group C (Table 1).

## 8. Intraoperative Parameters and Complications

The total time spent for operation was 1437.2 ± 563.1 s on average in all 352 patients. Mean phacoemulsification time taken and energy consumed in the procedure were 109.7 ± 77.3 s and 26.1 ± 20.0 mJ, respectively. There was no statistical significance in mean operation time, mean phacoemulsification time, and mean phacoemulsification energy between groups A, B, and C (Table 2).

During phacoemulsification, posterior capsular rupture is one of the most commonly seen intraoperative complications. In total, 2 (0.6%) cases of posterior capsular rupture were recorded in this study, with one case from group A and the other case from group C (Table 2).

## 9. Postoperative Outcomes of HCEC

The mean postoperative ECD was 1882. ± 691.8 in the whole 352 patients. The coefficient of variance and cell hexagonality were 37.5 ± 10.6% and 59.2 ± 11.7%, respectively. Combined with the preoperative ECD status, mean HCEC loss was 18.2 ± 12.3%. As for the cell morphology, the mean coefficient of variance change and mean cell hexagonality change values were 3.3 ± 11.0% and −4.5 ± 11.1%, respectively, and no statistical significance were observed. Interestingly, mean HCEC loss was 28.9 ± 9.2%, which was statistically significant higher in group B than in groups A and C (Table 3).

The postoperative CCT change was 9.4 ± 17.7 μm, with no statistically significant difference between the three groups. Likewise, the postoperative logMAR change in 1 month was −0.58 ± 0.54, with no statistically significant difference between three groups (Table 3).

## 10. Factors Associated with HCEC Loss after Phacoemulsification

Pearson’s chi-squared tests evaluated the correlation of postoperative ECD change percentage and factors related to patient’s condition and phacoemulsification. There was significant correlation in percentage of ECD change between age (*p =* 0.003), cataract grade (*p =* 0.005), phaco energy (*p <* 0.001), phaco time (*p =* 0.001), total operation time (*p =* 0.043), preoperative logMAR (*p =* 0.048), and preoperative ECD count (*p <* 0.001) (Table 1 and Table 2). A correlation was also shown between age and cataract grade (*p =* 0.006), phaco energy (*p <* 0.001), phaco time (*p =* 0.001), gender (*p =* 0.009), hypertension (*p =* 0.012), shallow AC (*p =* 0.005), preoperative IOP (*p =* 0.025), and postphaco HCEC hexa change (*p =* 0.003). A significant correlation was found between phaco energy and age (*p <* 0.001), total OP time (*p <* 0.001), phaco time (*p <* 0.001), cataract grade (*p <* 0.001), postoperative ECD count (*p <* 0.001), and 1-month and 6-month post OP logMAR change (*p =* 0.009, *p =* 0.010). Besides, preoperative CCT was found to be associated with postoperative logMAR change in the first month (*p =* 0.009) but not in the sixth month (*p =* 0.208).

Factors associated with postoperative ECD change were analyzed through multiple linear regression. In the independent variables of age, phaco energy, preoperative logMAR, and preoperative ECD number, the p-values were 0.034, <0.001, 0.037, and <0.001 respectively, showing a significant relationship with the dependent variable, percentage of postoperative ECD change (Table 4).

## 11. Discussion

Previously, higher ECD loss after phacoemulsification was described as being related to age [19], ocular comorbidities such as glaucoma [19], cornea guttata [20], ocular trauma [21], anterior chamber depth [22], eyes with occludable angles [12], cataract grade [23], or the inexperience of surgeons [11] (Table 4). O’Brien et al. reported a similar mean ECD loss rate of 11.6% in patients operated upon by less experienced surgeons as compared to more senior operators in our study [11]. Ko et al. demonstrated in eyes with occludable angles after phacoemulsification an ECD loss rate as high as 14.5%, and concluded that preoperative IOP, which was not a differential factor in our patients, was associated with the ECD loss rate [12]. Gogate et al. further analyzed different operation methods for ECD loss, and the study included mature cataracts, white cataracts, and up to grade 4 cataracts, which may lead to higher ECD loss rates (18.4%) [23]. Choi et al. conducted a long-term follow-up of more than 10 years after phacoemulsification and concluded that aging and bullous keratopathy may be responsible for increased ECD loss and compromised visual outcome. Despite the obvious increased ECD loss rate (20.6%), cell hexagonality and coefficient of variance were found to be similar after the long-term follow up [24]. Notably, all the aforementioned studies focused on patients with normal preoperative ECD levels.

Moreover, some studies excluded patients with mildly or markedly low ECD [11,23,24,25], while others focused on markedly low ECD [3,4] or had no exclusion criteria for ECD level [12], and showed no significant correlations between preoperative ECD and severity of ECD loss after phacoemulsification. In contrast to previous reports, we further divided our patients into three groups according to preoperative ECD levels and found higher risk for ECD loss in patients with mildly low ECD levels. Interestingly, in our data, preoperative ECD presented a normal distribution in group A and group C, but not in group B. The normality of ECD number decreased after phacoemulsification in group A and group B, but not in group C due to high skewness in group C (skewness = −1.16). As for the normality of ECD decrease, a percentage existed only in group A. The ECD was relatively stable in density at >2000 cells/mm^2^ (group C), and at <1000 cells/mm^2^ (group A). We assume that the ECD between 1000 and 2000 cells/mm^2^ represents a transition status between healthy and compromised status; thus, those HCECs are more vulnerable to the damage of phacoemulsification. The ECD distribution after phacoemulsification in this study was in a bimodal mode (Figure S1).

Besides ECD level, coefficient of variance and cell hexagonality were also used to evaluate corneal endothelial function clinically. In normal healthy adults, HCECs present as homogenous hexagonal cells. Once HCECs are lost due to aging, trauma, or other diseases, the remaining cells expand to fill other spaces due to the limited regenerative potential of HCEC. Thus, an increased coefficient of variance and decreased hexagonality would be observed [26,27]. The present study showed an increase of 3.3% with regard to mean of coefficient of variance change and a 4.5% decrease in hexagonality change in all patients after phacoemulsification. One study reported decreased HCEC hexagonality and increased coefficient of variance 1 month after cataract surgery [28], while another indicated decreased HCEC hexagonality with average endothelial cell size increases immediately after phacoemulsification, but not increased heterogenous HCECs [29]. Our results were consistent with the previous reports. With further analysis, no statistical significance was found for changes in HCEC hexagonality and the coefficient of variance among three groups.

Considering the lower ECD level, lower hexagonality, and higher coefficient of variation, HCECs in the markedly and mildly low ECD level group were indeed in a relatively compromised status compared to those in the normal ECD level group [29]. In general, HCECs would decrease spontaneously with aging regardless of preoperative HCEC status [30]. However, environmental stress, for example through cataract surgery, would accelerate the process of HCEC loss. Accordingly, we advocate preventive strategies such as the use of less phacoemulsification energy and earlier surgical interventions, as well as novel topical corneal endothelial protective agents for patients with mildly low ECD levels [31,32,33].

The condition of phacoemulsification has been proven to be a crucial intraoperative factor for postoperative ECD loss in previous studies [8,34,35]. Longer phacoemulsification time, higher phacoemulsification energy, high vacuum, and lower phacoemulsification frequency are independent parameters for increasing ECD loss [8,35]. In our results, phacoemulsification time and phacoemulsification energy were recorded and did not show significance statistically significant differences between these three groups, suggesting surgical environments of equal stress. Besides, phacoemulsification time and phacoemulsification energy were usually positively correlated to the density of nucleus, which is also an independent risk factor for ECD loss [8,34]. Our data suggested that phacoemulsification energy was a better risk factor to predict ECD loss after phacoemulsification compared to cataract grade (*p <* 0.001 vs. *p =* 0.005 in correlation analysis), similar to the findings pf Tsao et al. [16].

Diabetes mellitus is a common systemic disease worldwide. Except for diabetes retinopathy, major complications of ocular manifestation and other types of corneal dysfunction such as decreased ECD level, decreased HCEC hexagonality, increased coefficient of variance, and increased CCT were also reported [36,37]. In this study, 12 (40.0%) cases of diabetes mellitus were present in the markedly low ECD group, 23 (32.4%) cases in the mildly low ECD group, and 72 (28.6%) cases in the normal ECD group. In a large-scale systematic review, it was shown 1.4% to 18.4% non-diabetic and 6.2% to 29.7% diabetic patients had complications three months after cataract surgery [38] which is compatible with our results of 18.1 ± 11.6%.

Posterior capsular rupture is one of the most common complications of cataract surgery, and can lead to postoperative complications such as macular edema, endophthalmitis, or even bullous keratopathy. The prevalence of posterior capsular rupture varies according to different surgeons. Ocular risk factors include hard nucleus, shallow anterior chamber, previous ocular surgery, or concurrent ocular surgery [4,39]. In our results, only two cases in total (one case in group A and one case in group C) were found with posterior capsular rupture during the operation. Further follow-up of the two patients failed to detect subsequent postoperative complications.

Only one case of postoperative cornea edema in group C was found. However, we did not record subsequent corneal status indicators such as time to recovery or the need for another corneal transplantation operation. In addition, a reduced focus on postoperative corneal status, subsequent procedure-related complications, and the use other procedures were mentioned in other studies [4,9]. In future work, we will consider postoperative complications and the clinical courses of the patients.

There are several limitations in this study. First, as a retrospective study, not all clinical variances could be precisely determined, and some confounding bias could not be avoided due to different judgment of clinical physicians who recorded the chart. Second, for patients with ocular diseases such as Fuchs’ endothelial dystrophy or bullous keratopathy it was difficult to obtain precise ECD levels, which would cause bias. Third, the follow-up period differed from patient to patient depending on the condition and postoperative recovery.

In our study, patients with a preoperative ECD of 2268.7 ± 679.5 cells/mm^2^ were enrolled. Age, preoperative logMAR, cataract grade, preoperative ECD, operation time, phacoemulsification time, and phacoemulsification energy were significantly associated with postoperative ECD change (%). Gender, operation eye, hypertension, diabetes, glaucoma, corneal dystrophy, anterior chamber depth, preoperative IOP, CV, HEX, and CCT showed no significant relationships with the postoperative ECD change (%) change in statistics. We further analyzed the impact of preoperative ECD on postoperative ECD change (%) and found that the mild low ECD group (ECD between 1000 to 2000 cells/mm^2^) had a significantly decreased ECD as compared to the normal and low ECD groups, which has been discussed and may be a novel factor to predict ECD change after phacoemulsification. Less phacoemulsification energy, earlier surgical interventions, or novel topical corneal endothelial protective agents might be suggested for patients with ECD ranging from 1000 to 2000 cells/mm^2^.

## Figures and Tables

**Table 1:** Demographic information and ocular status of the 361 patients before phacoemulsification.

Parameter	Total	Chi-Squared Test *p* Value ^+^	Group A	Group B	Group C	One-Way ANOVA *p* Value
Participant number	352		29	71	252	
Age (year)						
Mean ± SD	69.0 ± 9.9	0.003 *	68.9 ± 10.0	69.6 ± 10.1	68.9 ± 9.9	0.841
Range	41–90		48–86	46–87	41–90	N/A
Gender		0.661				
Male	143 (40.6%)		9 (31.0%)	26 (36.6%)	108 (42.9%)	0.352
Female	209		20	45	114	N/A
Eye		0.416				
Right	181 (51.4%)		17 (58.1%)	39 (54.9%)	125 (49.6%)	0.528
Left	169		11	31	127	N/A
Bilateral	2		1	1	0	
Preoperative IOP (mmHg)						
Mean ± SD	15.0 ± 4.0	0.365	15.4 ± 4.2	14.6 ± 3.3	15.0 ± 4.1	0.570
Preoperative VA						
Mean logMAR ± SD	1.05 ± 0.47	0.048 *	0.93 ± 0.33	1.05 ± 0.45	1.05 ± 0.49	0.412
Cataract grade (%)		0.005 *				0.198
I	4 (1.1%)		0	1 (1.4%)	3 (1.2%)	
II	320 (90.9%)		29 (100%)	61 (85.9%)	230 (91.3%)	
III	28 (8.0%)		0	9 (12.7%)	19 (7.5%)	
ACD (mm)	2.78 ± 0.29	0.160	2.76 ± 0.38	2.81 ± 0.23	2.77 ± 0.30	0.551
Comorbidities						
Glaucoma	12 (3.4%)	0.766	0 (0%)	3 (4.2%)	9 (3.6%)	0.555
Corneal dystrophy	32 (9.1%)	0.182	24(82.1%)	4 (5.6%)	4 (1.6%)	<0.001 *
Hypertension	133 (37.8%)	0.326	11 (36.7%)	27 (38.0%)	95 (37.7%)	0.999
Diabetes mellitus	107(30.4%)	0.923	12 (40.0%)	23 (32.4%)	72 (28.6%)	0.338
Preoperative HCEC status						
Cell density ± SD(cell/mm^2^)	2268.7 ± 679.5	<0.001 *	814.2 ± 119.7	1512.0 ± 271.4	2646.1 ± 288.2	<0.001 *
CV %	34.2 ± 11.1	0.650	40.8 ± 13.9	36.4 ± 11.8	32.8 ± 10.2	<0.001 *
HEX %	63.8 ± 10.0	0.215	58.4 ± 14.6	60.3 ± 11.9	65.3 ± 8.1	<0.001 *
CCT (μm) Mean ± SD	553.3 ± 34.0	0.981	557.8 ± 35.7	557.6 ± 36.1	551.0 ± 33.7	0.269

Demographic data were collected and analyzed from all patients. * *p*-value < 0.05. **^+^** The chi-squared test was used to examine the association between factors and postoperative ECD change (%). ACD: anterior chamber depth; CV: coefficient of variation; CCT: central cornea thickness; IOP: intraocular pressure; HCEC: human corneal endothelial cells; HEX: hexagonality; logMAR: logarithm of the minimum angle of resolution. Group A: Patient preoperative endothelial cell density (ECD) below 1000 cells/mm^2^. Group B: Patient preoperative endothelial cell density (ECD) between 1000 and 2000 cells/mm^2^. Group C: Patient preoperative endothelial cell density (ECD) above 2000 cells/mm^2^.

**Table 2:** Intraoperative features among groups according to preoperative corneal ECD.

Parameters	Total	Chi-Square Test *p* Value ^+^	Group A	Group B	Group C	One-Way ANOVA *p* Value
Participant number	352		29	71	252	
Time spent (s)						
Mean OP time	1437.2 ± 563.1	0.043 *	1553.3 ± 340.3	1417.1 ± 372.2	1429.5 ± 624.5	0.505
Mean Phaco time	109.7 ± 77.3	0.001 *	108.9 ± 43.7	102.6 ± 62.3	111.9 ± 83.9	0.697
Phaco energy (mJ)	26.1 ± 20.0		21.8 ± 10.1	28.4 ± 17.4	26.0 ± 21.5	0.323
Mean ± SD		<0.001 *				
PC rupture (%)	2 (0.6%)		1 (3.4%)	0 (0.0%)	1 (0.4%)	

* *p*-value < 0.05. ^+^ The chi-squared test was used to examine the association between factors and postoperative ECD change (%). OP: operation; Phaco: phacoemulsification; PC: posterior capsular. Group A: Patient preoperative endothelial cell density (ECD) below 1000 cells/ mm^2^. Group B: Patient preoperative endothelial cell density (ECD) between 1000 and 2000 cells/mm^2^. Group C: Patient preoperative endothelial cell density (ECD) above 2000 cells/mm^2^.

**Table 3:** Corneal and visual outcomes among groups according to preoperative corneal ECD.

Characteristics	Total	Group A	Group B	Group C	*p* Value
Participant number	352	29	71	252	
Postoperative HCEC status					
Cell density ± SD cell/mm^2^	1882 ± 691.8	675.4 ± 112.3	1063.5 ± 179.2	2251.5 ± 405.1	<0.001 *
Cell variance ± SD	37.5 ± 10.6	43.1 ± 13.1	39.5 ± 11.5	36.3 ± 9.7	<0.001 *
Cell hexagonality ± SD	59.2 ± 11.7	54.5 ± 15.3	56.0 ± 11.9	60.7 ± 10.9	0.001 *
CCT (μm) ± SD	562.7± 33.9	558.8± 35.9	557.6± 36.1	551.5± 33.0	0.133
^a^ ECD change (%) ± SD	−18.2 ± 12.3	−19.9 ± 5.4	−28.9 ± 9.2	−15.0 ± 12.0	<0.001 *
^b^ HCEC CV change	3.3 ± 11.0	2.7 ± 13.2	3.1 ± 11.4	3.4 ± 10.7	0.941
^c^ HCEC hexa. change	−4.5 ± 11.1	−3.9 ± 17.2	−4.4 ± 10.5	−4.7 ± 10.5	0.937
CCT change (μm)					
Mean ± SD	9.4 ± 17.7	13.8 ± 14.4	8.3 ± 17.5	9.2 ± 18.0	0.346
First month postoperative logMAR change, Mean ± SD (n)	−0.58 ± 0.54 (327)	−0.54 ± 0.45 (28)	−0.52 ± 0.51 (66)	−0.60 ± 0.56 (234)	0.557

* *p*-value < 0.05. CCT: central corneal thickness; CV: cell variance; ECD: endothelial cell density; hexa: hexagonality; logMAR: logarithm of the minimum angle of resolution. Group A: Patient preoperative endothelial cell density (ECD) below 1000 cells/mm^2^. Group B: Patient preoperative endothelial cell density (ECD) between 1000 and 2000 cells/mm^2^. Group C: Patient preoperative endothelial cell density (ECD) above 2000 cells/mm^2^. ^a^ ECD change (%) = (preoperative cell density − postoperative cell density)/preoperative cell density. ^b^ HCEC CV change = postoperation CV − preoperation CV. ^c^ HCEC hexa change = postoperation hexa − preoperation hexa.

**Table 4:** Parameters estimated through multiple linear regression analysis.

Coefficients ^a^							
Model	Unstandardized Coefficients		Standardized Coefficients	t	Sig.	Collinearity Statistics	
	B	Std. Error	Beta			Tolerance	VIF
Constant	−13.420	5.167		−2.597	0.010		
Age	−0.190	0.062	−0.153	−3.052	0.002	0.979	1.021
Phaco energy	−0.001	0.002	−0.024	−0.479	0.632	0.991	1.009
Preoperative logMAR	−4.495	1.317	−0.171	−3.412	0.001	0.978	1.022
Preoperative ECD	0.006	0.001	0.318	6.374	0.000	0.986	1.014

^a^. Dependent variable: ECD_change (%). ECD: endothelial cell density; logMAR: logarithm of the minimum angle of resolution; phaco: phacoemulsification.

## Supplementary Materials

The following are available online at https://www.mdpi.com/article/10.3390/jcm10112270/s1, Figure S1: Histogram of pre- and post-operatively ECD count in this study, Table S1: Normality test of pre- and post-operatively ECD in subgroups.
