# Supplementary material for: Accelerated Corneal Endothelial Cell Loss after Phacoemulsification in Patients with Mildly Low Endothelial Cell Density
Source: J Clin Med. 2021 May 24;10(11):2270. doi: 10.3390/jcm10112270 (PMC8197237; doi:10.3390/jcm10112270)
Supplement: Supplementary file 1 [file jcm-10-02270-s001.zip › jcm-1220769-supplementary.pdf]

Supplementary Materials:

Figure S1. Histogram of pre- and post-operatively ECD count in this study.

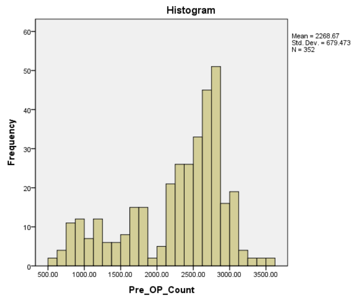

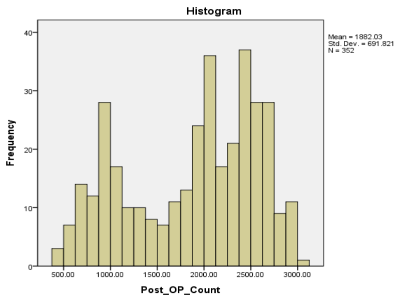

Table S1. Normality test of pre- and post-operatively ECD in subgroups.

Tests of Normality	Group	Kolmogorov-Smirnova			Shapiro-Wilk		
		Statistic	df	Sig.	Statistic	df	Sig.
Pre_OP_Count	A	0.115	29	0.200*	0.936	29	0.077
	B	0.119	71	0.014	0.933	71	0.001
	C	0.042	252	0.200*	0.982	252	0.003
Post_OP_Count	A	0.107	29	0.200*	0.956	29	0.257
	B	0.143	71	0.001	0.951	71	0.008
	C	0.075	252	0.002	0.974	252	0.000

* This is a lower bound of the true significance.
